# Epidemiology, clinical features, and management of severe hypercalcemia in critically ill patients

**DOI:** 10.1186/s13613-019-0606-8

**Published:** 2019-11-27

**Authors:** Cyril Mousseaux, Axelle Dupont, Cédric Rafat, Kenneth Ekpe, Etienne Ghrenassia, Lionel Kerhuel, Fanny Ardisson, Eric Mariotte, Virginie Lemiale, Benoît Schlemmer, Elie Azoulay, Lara Zafrani

**Affiliations:** 1 Medical Intensive Care Unit, Saint-Louis Hospital, Assistance Publique-Hopitaux de Paris, Paris Diderot University, Paris, France; 2 Biostatistics Department, Saint-Louis Hospital, Assistance Publique-Hopitaux de Paris, Paris Diderot University, Paris, France; 3 Nephrology Department, Tenon Hospital, Assistance Publique-Hopitaux de Paris, Paris Sorbonnes University, Paris, France

**Keywords:** Hypercalcemia (HCM), Onco-hematology, Acute kidney injury (AKI), AKI etiology, Renal replacement therapy (RRT), Kidney disease outcome, Prognosis, Mortality

## Abstract

**Background:**

Severe hypercalcemia (HCM) is a common reason for admission in intensive-care unit (ICU). This case series aims to describe the clinical and biological features, etiologies, treatments, and outcome associated with severe HCM. This study included all patients with a total calcemia above 12 mg/dL (3 mmol/L) admitted in two ICUs from January 2007 to February 2017.

**Results:**

131 patients with HCM were included. HCM was related to hematologic malignancy in 58 (44.3%), solid tumors in 29 (22.1%), endocrinopathies in 16 (12.2%), and other causes in 28 (21.3%) patients. 108 (82.4%) patients fulfilled acute kidney injury (AKI) criteria. Among them, 25 (19%) patients required renal replacement therapy (RRT). 51 (38.9%) patients presented with neurological symptoms, 73 (55.7%) patients had cardiovascular manifestations, and 50 (38.1%) patients had digestive manifestations. The use of bisphosphonates (HR, 0.42; 95% CI, 0.27–0.67; *P *< 0.001) was the only treatment significantly associated with a decrease of total calcemia below 12 mg/dL (3 mmol/L) at day 5. ICU and Hospital mortality rates were, respectively, 9.9% and 21.3%. Simplified Acute Physiologic Score (SAPS II) (OR, 1.05; 95% CI 1.01–1.1; *P* = 0.03) and an underlying solid tumor (OR, 13.83; 95% CI 2.24–141.25; *P* = 0.01) were two independent factors associated with hospital mortality in multivariate analysis.

**Conclusions:**

HCM is associated with high mortality rates, mainly due to underlying malignancies. The course of HCM may be complicated by organ failures which are most of the time reversible with early ICU management. Early ICU admission and prompt HCM management are crucial, especially in patients with an underlying solid tumor presenting with neurological symptoms.

## Background

Hypercalcemia (HCM) is commonly defined by a serum calcium level above 2.6 mmol/L or 10.5 mg/dL, 40% of which is ionized calcium [1]. Despite the lack of a clear definition of a severe HCM, a calcemia above 12 mg/dL (3 mmol/L) is frequently retained.

Because HCM may lead to life-threatening complications, patients often require Intensive-Care Unit (ICU) admission. Clinical symptoms are non-specific, depending on calcium levels and rapidity of onset. They include renal complications, ranging from polyuria to acute kidney injury (AKI) [2]; cardiovascular complications, including sinus tachycardia, hypertension, and infarct-like ST segment elevation [3]; digestive events, from abdominal pain to acute pancreatitis [4], and neurological impairments, including seizures [5], delirium, and coma.

Epidemiological data focusing on HCM in ICU are scarce. In a multicentric retrospective study, severe hypercalcemia (defined by a ionized calcemia > 2.9 mEq/L or 1.45 mmol/L) occurred in 2% of critically ill patients [6]. Few studies found an independent association between HCM and hospital or ICU mortality [7, 8].

Among all causes of HCM, primary hyperparathyroidism and malignancies (including solids tumor and hematologic malignancies) are predominant. Recent epidemiological studies estimate that HCM affects between 0.65 and 3% of all oncology patients [9, 10]. Within tumor-related etiologies, multiple myeloma, breast, lung, and kidney cancers are the most frequent. In this context, HCM often occurs at a metastatic stage [9], which is no more per se a contraindication for ICU admission [11, 12]. However, there are no data available on etiologies responsible for HCM in ICU patients.

Due to the scarcity of epidemiological and clinical data of patients suffering from HCM in ICU, we conducted a retrospective study of 131 patients in two different French ICUs. Our objectives were to characterize clinical and biological features of HCM, to identify risk factors for complications of HCM and risk factors of mortality.

## Methods

This present work is a retrospective study performed in two distinct ICUs. The medical ICU of the Saint-Louis University Hospital, Paris, France, is a 12-bed medical unit that admits 850 patients per year, of whom about one-third have hematologic malignancies. The nephrology ICU of the Tenon University Hospital, Paris, France is a 17-bed medical unit that admits 900 patients per year. The study was approved by the ethical committee of the “Société de reanimation de langue française” (n°18-34).

### Patients

We included all adult patients admitted in ICU with severe HCM from January 2007 through February 2017. We defined severe HCM by a total calcemia above 12 mg/dL (3 mmol/L) [13, 14]. When needed, calcemia was corrected by serum albumin level, and in default of, by total protein levels.

### Definitions

Definition and staging of AKI were defined according to the 2012 KDIGO (Kidney Disease: Improving Global Outcomes) guideline [15]. Decisions regarding the initiation, discontinuation, and modalities of renal replacement therapy (RRT) were made by senior nephrologists based on the guidelines from Bellomo and Ronco [16].

Etiologies of HCM were retrospectively subdivided in four groups: (1) hematologic malignancies; (2) solid tumors; (3) endocrinopathies; (4) other causes, including iatrogenic, granulomatosis, and unknown causes. We sought for etiological investigations including the testing of PTH or 1–25(OH) Vit D during the hospital stay.

Based on known complications of HCM [1, 17], we classified HCM manifestations in four entities: (1) renal manifestations, including polyuria and acute kidney injury; (2) cardiovascular manifestations, including de novo arterial hypertension and any EKG manifestations; (3) neurological manifestations, including seizures and mental status alteration; and (4) digestive manifestations, such as acute pancreatitis, digestive occlusion, or constipation.

We used the Glasgow scale score [18] for evaluation of mental status at admission.

Bisphosphonate safety was assessed on creatinine variation 3 months after admission in ICU.

### Patient’s characteristics

Demographic parameters, medical history, presenting symptoms, and treatments were collected. All laboratory data were recorded at admission. Serum creatinine level was recorded 3 months before ICU admission when possible, at ICU admission, ICU discharge, hospital discharge, 3 and 6 months after ICU and at last follow-up. Sequential Organ Failure Assessment (SOFA) and Simplified Acute Physiology Score (SAPS II) parameters were collected on day 1 [19, 20]. ECOG/WHO performance status [21], and Charlson comorbidity index [22] were evaluated based on precedent medical records.

Serum PTH level were measured using a radio-immunologic assay (CIS-Bio-Radio-ImmunoAnalysis^®^) with a normal range between 8 and 76 ng/L. Serum 25-dihydroxyvitamin D was measured using a radio-immunologic method (Diasorin^®^) that recognized both 25-hydroxyvitamin D2 and 25-hydroxyvitamin D3. An excess of 25-dihydroxyvitamin D was arbitrary defined by a level above 100 ng/mL. Serum 1,25-dihydroxyvitamin D was measured using a radio-immunologic method (Diasorin^®^) with reference values ranging from 20 to 60 pg/mL. Plasma phosphate was measured using colorimetry (phosphomolybdate assay) and serum ionized calcium, using a specific electrode.

Vital status at ICU discharge, hospital discharge, and last follow-up were determined from medical records and the outpatient clinic electronic database.

### Treatment

All patients received the standard of care. The period and duration of renal replacement therapy were collected. Bisphosphonate treatment dose and duration were recorded. Type and daily volume of hydration, corticosteroid treatment, use of salmon calcitonin, furosemide in hypocalcemic purpose, and calcimimetic were collected.

### Statistical analyses

Patients’ characteristics at ICU admission are described as median and interquartile range (IQR) for quantitative variables and frequencies and percentages for qualitative variables. Distribution of baseline variables, life-supporting treatments, and specific treatments of HCM were compared between patients alive or not at leaving the ICU using the Wilcoxon test for quantitative variables, and Fisher’s test for qualitative variables.

Cumulative incidence of mortality in ICU and hospital was estimated, taking ICU or hospital discharge as competing risk. Univariate analysis for complications was performed in logistic regression to identify factors associated with complications. For mortality risk in ICU, univariate analysis with logistic regression was adjusted on SAPSII scale score and Charlson morbidity index. Multivariate models were adjusted on age, SAPSII for mortality, pre-existing cardiopathy for cardiovascular complications, CKD for AKI stage 3, and variables that were significant at 0.15 level.

The endpoint of treatment analysis was the diminution of total calcemia below 3 mmol/L (12 mg/dL) between day 0 and day 5. Univariate analyses were performed in Cox proportional hazards regression models. Final multivariate model was adjusted on etiologies and significant treatments at 0.15 levels. Patients with stage V-CKD were excluded from this analysis. All tests were two sided with a 5% type I error. All calculations were performed with the R software (version 3.4.4)^®^.

## Results

### Patients’ characteristics at ICU admission

During the study period, 131 patients presented with severe hypercalcemia. Patient characteristics at ICU admission are reported in Table 1. Median SOFA score and SAPSII score were 2 (IQR, 1; 4) and 29.5 (IQR, 22; 36.75), respectively. HCM was related to hematologic malignancies in 58 (44.3%) patients, solid tumors in 29 (22.1%) patients, endocrinopathies in 16 (12.2%) patients, and other causes in 28 (21.3%) patients. Endocrinopathies were mainly due to primary hyperparathyroidism in 15 (93.7%) patients. Other causes are detailed in Table 1. Among them, 15 patients had a suspected vitamin D intoxication [14 received 1–25 (OH) vitamin D and 4 received cholecalciferol for 25 (OH) vitamin D supplementation]. In most cases (14/15), patients received additional calcium supplementation. 14/15 patients had a past of thyroidectomy with 7 post-surgery hypoparathyroidism and 1 hypocalcemia of unknown origin. Patients’ characteristics with suspected vitamin D intoxication are summarized in Additional file 1: Table S1. HCM was the main reason for ICU admission in 101 (77.1%) patients.

**Table 1:** Patients’ demographic and clinical characteristics at admission

	All (*n* = 131)	Hemopathy (*n* = 58)	Solid tumors (*n* = 29)	Endocrinopathies (*n* = 16)	Other (*n* = 28)
Clinical data					
Age (y)	58.5 [48.5; 69]	58 [42; 67]	63 [52; 68]	52 [36.5; 67.25]	66 [55.25; 74.25]
Women, *n* (%)	64 (48.8)	22 (38)	17 (58.6)	9 (56)	16 (57.1)
Chronic kidney disease, *n* (%)	25 (19.5)	10 (17.2)	2 (6.8)	4 (25)	10 (35.7)
Cardiopathy, *n* (%)	32 (25)	8 (13.7)	6 (20.7)	4 (25)	15 (53.5)
SOFA score at day 1	2 [1, 4]	4 [2, 5]	1 [1, 3]	1 [0; 2]	2 [1, 3]
SAPS II score at day 1	29.5 [22; 36.75]	34 [28, 40]	33 [24, 37]	19.5 [16.75; 29.25]	27 [18.5; 33]
ECOG/WHO scale score	1 [0; 3]	1 [0; 2]	3 [1, 3]	0.5 [0; 2.75]	1 [0; 3]
Charlson score	4 [2, 6]	3 [2, 4]	7 [6, 9]	3 [1, 6]	4 [2; 6.25]
Admission diagnosis in ICU					
Hypercalcemia, *n* (%)	101 (77.1)	42 (72.4)	22 (76)	13 (81)	23 (82)
Coma, delirium, *n* (%)	9 (6.8)	3 (5.2)	5 (17.2)	0	1 (3.5)
Acute respiratory failure, *n* (%)	5 (3.8)	4 (6.8)	1 (3.4)	0	0
Tumor lysis syndrome, *n* (%)	4 (3)	4 (6.8)	0	0	0
Acute kidney injury, *n* (%)	4 (3)	2 (3.4)	1 (3.4)	0	1 (3.5)
Sepsis, *n* (%)	5 (3.8)	1 (1.7)	0	3 (19)	1 (3.5)
Other, *n* (%)	3 (2.2)	2 (3.4)	0	0	2 (7)

Values for categorical variables are given as number (percentage); values for continuous variables, as median [interquartile range]

Other causes included iatrogenic causes (*n* = 16; 57.1%), sarcoidosis (*n* = 4; 14.2%), tuberculosis (*n* = 1; 3.5%), atypical mycobacterial infection (*n* = 1; 3.5%), global dehydration (*n* = 1; 3.5%), and unknown causes (*n* = 5; 17.8%)

*ECOG/WHO* Eastern Cooperative Oncology Group/World Health Organization, *SAPS II* Simplified Acute Physiology Score, *SOFA* Sequential Organ Failure Assessment

### Clinical features

**Fig. 1:**
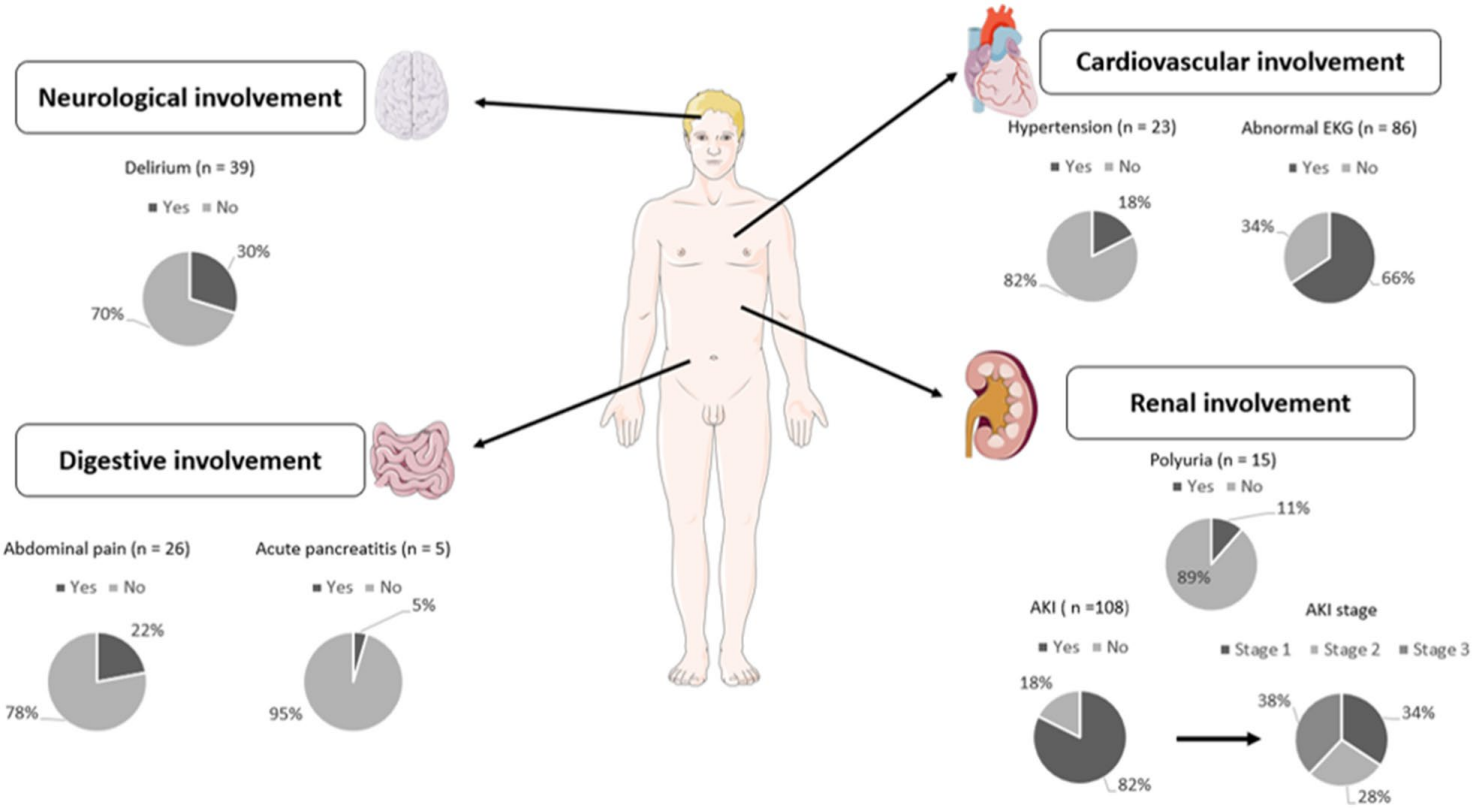
Clinical manifestations of HCM. *AKI* acute kidney injury, *AKI* stage based on KDIGO guidelines, *EKG* electrocardiogram

Patients’ clinical features are reported in Fig. 1 and renal manifestations are reported in Table 2. Fifteen (11.4%) patients had polyuria before ICU admission. One hundred and eight (82.4%) patients fulfilled AKI criteria: stage 1, 37 (34.2%); stage 2, 30 (27.7%); and stage 3, 41 (37.9%). The main presumed diagnoses for AKI were hypoperfusion (63%), acute tubular necrosis (19%), cast nephropathy (16%), obstructive (10%), and tumor lysis syndrome (7%). Thirty (23%) patients presented multiple possible causes of AKI. No patient had a kidney biopsy during ICU stay.

**Table 2:** Characteristics of AKI

Variable	Value
Scr (mg/dL) 3 months before ICU admission	0.9 [0.7; 1.2]
eGFR (mL/min/1.73 m^2^) before ICU admission	85 [63; 109]
Polyuria, *n* (%)	15 (11.4)
AKI, *n* (%)	108 (82.4)
AKI stage	
Stage 1, *n* (%)	37 (34.2)
Stage 2, *n* (%)	30 (27.7)
Stage 3, *n* (%)	41 (37.9)
Scr (mg/dL) at ICU admission	1.88 [1.1; 3.2]
Scr (mg/dL) at day 3	1.5 [0.9; 2.7]
Scr (mg/dL) at day 5	1.4 [0.9; 2.5]
Scr (mg/dL) at day 7	1.3 [0.8; 2.3]
Maximal Scr (mg/dL)	1.95 [1.1; 3.4]
Scr (mg/dL) at ICU discharge	1 [0.7; 1.7]
RRT during ICU stay	25 (19%)
Duration of RRT (d)	5 [2.5; 7]
Causes of AKI	
Hypoperfusion	68 (63%)
Acute tubular necrosis	20 (19%)
Cast nephropathy	18 (16%)
Obstructive	11 (10%)
Tumor lysis syndrome	8 (7%)
Sarcoidosis	3 (3%)
Nephrotoxic agents	2 (2%)
Kidney infiltration by malignancy	1 (1%)
Amyloidosis	1 (1%)
> 1 cause	30 (23%)
Follow-up data	
Scr (mg/dL) at month 3 in RRT-free patients	0.9 [0.7; 1.3]
eGFR (mL/min/1.73 m^2^) at month 3 in RRT-free patients	86 [63; 109]
Scr (mg/dL) at month 6 in RRT-free patients	0.9 [0.7; 1.3]
eGFR (mL/min/1.73 m^2^) at month 6 in RRT-free patients	77 [54; 96]

n = 131. Percentages values are given based for the 108 patients who presented AKI. Values of categorical variables are given as number (percentage); values for continuous variables, as median [interquartile range]. Conversion factor for Scr in mg/dL to µmol/L is 88.4

*AKI* acute kidney injury, *eGFR* estimated glomerular filtration rate based on MDRD formula, *ICU* intensive-care unit, *RRT* renal replacement therapy, *Scr* serum creatinine

Conversion factor: Scr, serum creatinine in mg/dL to µmol/L, ×88.4

Details of extra-renal manifestations are reported in Additional file 1: Table S2. Fifty-one (38.9%) patients presented with neurological manifestations, mostly delirium (39 patients representing 29.8% of the total population). Seventy-three (55.7%) patients had cardiovascular manifestations. De novo arterial hypertension was noted in 23 (17.5%) patients. The most frequent EKG signs, reread by a trained senior, were sinus tachycardia (*n* = 31, 23.6%), conduction disturbances (*n* = 20, 15.2%), and short QT interval (*n* = 19, 14.5%). Fifty (38.1%) patients had digestive manifestations including abdominal pain in 26 (19.8%) patients.

### Calcemia course

Characteristics of calcemia levels and etiological investigations are reported in Table 3. Calcemia course between day 1 and day 9 is depicted in Additional file 1: Figure S1. Median total calcemia level at admission was 14.5 (IQR, 13.2; 16.3) mg/dL. Median magnesium level was 0.39 mEq/L (IQR, 0.34; 0.43) and median natremia was 138 mmol/L (IQR, 134; 140). Patients with HCM due to endocrinopathies had significative higher PTH median level (460 ng/mL, IQR, 197.5; 589 versus 8.5, IQR: 3.9; 16.25; P < 0.0001). As expected, patients with primary hyperparathyroidism tend to have a lower median level of phosphoremia (2.4, IQR, 1.9; 2.6 mg/dL versus 3.7, IQR, 2.8; 4.9 mg/dL, P < 0.0001) when compared with other patients. Fifty-two (39.7%) patients were tested for 1–25(OH) vitamin D with a median level at 16 pg/mL (IQR, 6.9; 28.3). Twenty-one patients (16%) had high 1,25-dihydroxyvitamin D levels (above 60 pg/mL). Among 65 (49.6%) patients tested for 25(OH) vitamin D, only 2 (3%) had an intoxication due to massive vitamin D ingestion. Diagnosis of paraneoplastic hypercalcemia with high PTH-rp level was retained in three (2.3%) patients.

**Table 3:** Calcemia course and etiological investigations of patients with hypercalcemia

	All (*n* = 131)
Calcemia	
Calcemia at day 1 (mg/dL)	14.5[13.2;16.3]
Corrected calcemia at day 1 (mg/dL)	15.6 [14;17.2]
Ionized calcemia at day 1 (mEq/L)	3.5 [3.1;3.9]
Minimal etiological screening	86 (65.6)
PTH: patients tested, *n* (%)	84 (64.1)
PTH median level (ng/mL)	11 [4.9; 47.5]
High PTH, *n* (%)	16 (19)
Low or abnormally normal PTH, *n* (%)	68 (81)
PTH-rp: patients tested, *n* (%)	17 (13)
High PTH-rp, *n* (%)	3 (2.3)
Active Vitamin D (1,25-dihydroxyvitamin D): patients tested, *n* (%)	52 (39.7)
Active Vitamin D (1,25-dihydroxyvitamin D) (pg/mL)	16 [6.9; 28.3]
High active Vitamin D (1,25-dihydroxyvitamin D) levels, *n* (%)	21 (16)
Vitamin D (25-dihydroxyvitamin D): patients tested, *n* (%)	65 (49.6)
Vitamin D (25-dihydroxyvitamin D) (ng/mL)	9.3 [5.8; 13.6]
High Vitamin D (25-dihydroxyvitamin D) levels, *n* (%)	2 (1.5)
Phosphatemia at admission (mg/dL)	3.5 [2.6; 4.9]

Values for categorical variables are given as number (percentage); values for continuous variables, as median [interquartile range]

Conversion factors: active Vitamin D (1,25-dihydroxyvitamin D) pg/mL for pmoL/L, ×2.6, Vitamin D (25-dihydroxyvitamin D) ng/mL for nmol/L, ×2.496, total calcemia in mg/dL for mmol/L, ×0.2495, calcium ion in mEq/L for mmol/L, ×0.5, phosphorus in mg/dL for mmol/L, ×0.3229

Normal range: PTH: between 5 and 60 pg/mL

*PTH* parathyroid hormone, *PTH-rp* parathyroid hormone-related protein

### Treatment characteristics and outcomes

Treatment characteristics and outcomes are detailed in Table 4.

**Table 4:** Treatment’s characteristics and outcomes

	All (*n* = 131)	Hemopathy (*n* = 58)	Solid tumors (*n* = 29)	Endocrinopathies (*n* = 16)	Other (*n* = 28)
Hyperhydration, *n* (%)	124 (94.6)	53 (91.3)	29 (100)	16 (100)	26 (92.8)
Crystalloid, *n* (%)	119 (90)	50 (87.7)	29 (100)	16 (100)	24 (85.7)
Volume at day 1 (L/day)	3 [2, 3]	3 [2, 3]	3 [2.5; 3]	3.25 [2.25; 4]	2 [2, 3]
Side effect: pulmonary edema, *n* (%)	11 (8.3)	9 (17)	2 (6.8)	0	0
Bisphosphonate infusion, *n* (%)	103 (78.6)	49 (84)	26 (89.6)	12 (75)	17 (60.7)
Second infusion, *n* (%)	13 (10)	5 (10.2)	5 (19.2)	0	3 (10.7)
Corticosteroids, *n* (%)	65 (50)	49 (84.4)	10 (34.5)	0	6 (21.4)
Furosemide, *n* (%)	13 (10)	6 (10.3)	1 (3.4)	0	6 (21.4)
Calcimimetics, *n* (%)	6 (5)	0	1 (3.4)	5 (31)	0
Calcitonin, *n* (%)	12 (9)	4 (6.8)	5 (17.2)	1 (6.3)	2 (7.1)
Renal replacement therapy, *n* (%)	25 (19)	20 (34.4)	3 (10.3)	1 (6.3)	1 (3.6)
Duration of RRT (d)	5 [2.5; 7]	5 [2.5; 6]	3	3	11
Vasopressor, *n* (%)	6 (4.5)	5 (8.6)	0	0	1 (3.5)
Mechanical ventilation, *n* (%)	10 (7.6)	6 (10.3)	3 (10.3)	0	1 (3.5)
ICU mortality, *n* (%)	13 (9.9)	4 (6.8)	6 (20.6)	1 (6.3)	2 (7.1)
Hospital mortality, *n* %)	26 (21.3)	10 (17.2)	13 (44.8)	1 (6.3)	2 (7.1)

Values for categorical variables are given as number (percentage); values for continuous variables, as median [interquartile range]

*ICU* intensive-care unit, *RRT* renal replacement therapy

One hundred and twenty-four (94.6%) patients received fluid administration, mainly with crystalloids. The main side effect was pulmonary edema in 11 (8.3%) patients. Bisphosphonates were used in 103 (78.6%) patients, especially in case of solid tumors and hematologic malignancy. Fifteen patients (14.5%) received bisphosphonates before ICU admission. Of the 103 patients who received bisphosphonates, 46 received zoledronate (4 mg, IQR, 4; 4), 47 patients received pamidronate (90 mg, IQR, 60; 90), and 6 patients received ibandronate (2 mg, IQR, 2; 3.5). Only 13 (10%) patients required a second infusion of bisphosphonates. Corticosteroids were administered in 65 (50%) patients. Most of them had an underlying hematologic malignancy (84.4% of the total). Only 13 (10%) patients received furosemide and 12 (9%) patients received calcitonin. No patient received denosumab. Twenty-five (19% of the total population and 23.1% of the population with AKI) patients needed RRT during their ICU stay. The median duration time was 5 days (IQR, 2.5; 7). Tumor lysis syndrome was the main indication for dialysis in 11 (8.4%) patients, followed by hypercalcemia in 6 (4.6%) patients and pulmonary edema in 4 (3%) patients. Vasopressors were used in 6 (4.5%) patients and mechanical ventilation needed in 10 (7.6%) patients. ICU mortality and hospital mortality rate were 9.9% and 21.3%, respectively.

### Risk factors for hypercalcemia-induced complications

Univariate analysis of risk factors associated with hypercalcemia-induced complications is shown in Additional file 1: Table 3. By multivariate analysis (Table 5), male sex (OR, 0.38; 95% CI 0.17–0.83; *P *= 0.02) was less associated with cardiovascular complications. Two factors were associated independently with neurological complications by multivariate analysis: total calcemia at day 1 (OR, 2; 95% CI 1.06–3.99; *P* = 0.04) and underlying solid malignancy (OR, 10.6; 95% CI 3.16–40.84; *P* < 0.01).

**Table 5:** Multivariate analysis of determinants of HCM complications

	OR (95% IC)	*P* value
Associated factors with cardiovascular complications		
Age > 60 years	2.05 (0.96;4.5)	0.07
Male sex	0.36 (0.16; 0.75)	0.01
Pre-existing cardiopathy	0.63 (0.26; 1.5)	0.3
Calcemia at day 1	1.68 (0.93; 3.32)	0.11
Associated factors with neurological complications		
Age > 60 years	1.89 (0.84; 4.41)	0.13
Calcemia at day 1	2 (1.06; 3.99)	0.04
Etiologies		
Other	1	
Hemopathies	2.48 (0.87; 7.9)	0.1
Solid tumors	10.58 (3.16; 40.84)	< 0.01
Endocrinopathies	0.6 (0.08; 3.16)	0.58
Associated factors with AKI stage > 2^a^		
Age > 60 years	0.3 (0.05; 1.65)	0.18
Etiologies		
Hemopathies	1	
Solid tumors	0.14 (0.013; 1.63)	0.12
Endocrinopathies and other causes	0.1 (0.009; 1.1)	0.06
Chronic kidney disease	1.31 (0.27; 6.37)	0.74

^a^Three patients were excluded due to pre-existing renal replacement therapy

*AKI* acute kidney injury

### Impact of HCM on mortality

**Fig. 2:**
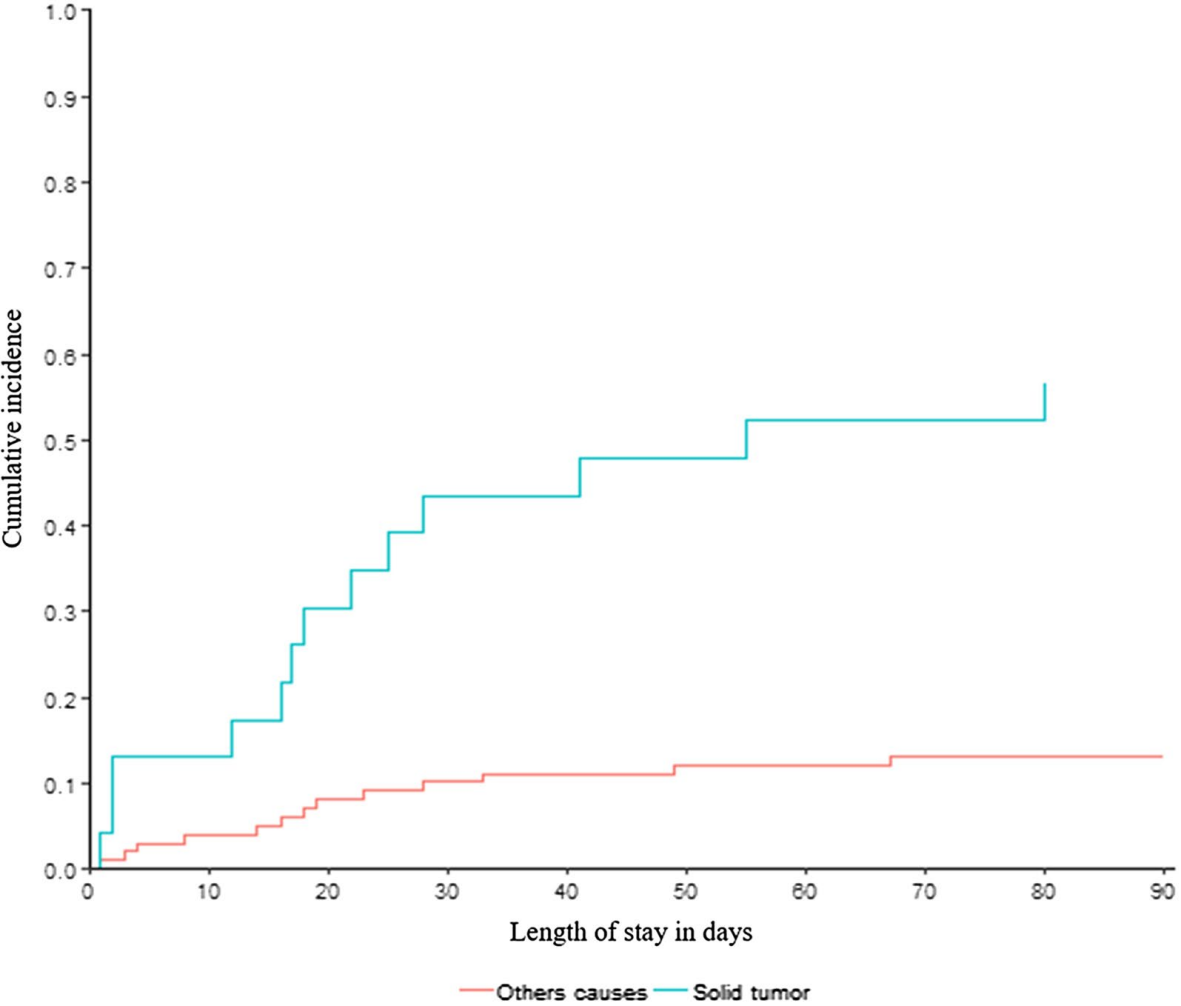
Cumulative incidence of hospital mortality based on solid tumor status. *Blue curve* HCM from tumor etiology. *Red curve* HCM from other causes

Figure 2 is the cumulative incidence curve of hospital mortality based on solid tumor etiology. In univariate analysis (Additional file 1: Table S3) adjusted for SAPSII scale score and Charlson morbidity index, solid tumors and neurological complications were associated with higher hospital mortality. We did not found an association between hospital mortality and bisphosphonate administration. Two factors were independently associated with higher mortality in multivariate analysis (Table 6): SAPSII scale score (OR, 1.05; 95% CI 1.01–1.1; *P* = 0.03) and underlying solid malignancy (OR, 13.83; 95% CI 2.24–141.25; *P* = 0.01).

**Table 6:** Multivariate analysis of determinants of hospital mortality

	OR (95% IC)	*P*
SAPSII scale score	1.05 (1.01;1.1)	0.03
Age > 60 years	2.17 (0.65;7.54)	0.21
Etiologies		
Others	1	
Solid tumors	13.83 (2.24; 141.25)	0.01
Endocrinopathies	1.81 (0.07; 24.97)	0.67
Hemopathies	2.76 (0.51; 24.51)	0.28
Neurological complications	2.60 (0.84; 8.36)	0.1

### Impact of treatment

We next analyzed the effectiveness of therapies (i.e., bisphosphonates, corticosteroids, furosemide, and calcitonin) to decrease hypercalcemia below 12 mg/dL (3 mmol/L) at day 5 (Additional file 1: Table 4). The use of bisphosphonates (HR, 0.42; 95% IC, 0.27–0.67; *P *< 0.001) was the only treatment significantly associated with a decrease of total calcemia below 12 mg/dL (3 mmol/L) at day 5. No difference was observed between ibandronate (HR = 0.79; IQR = [0.31–2.02], p = 0.62), pamidronate (HR = 0.71; IQR = [0.45–1.14], p = 0.16), and zoledronate. We did not found any association between worsened renal function at 3 months with bisphosphonates administration (p = 0.25). Twelve patients did not received bisphosphonates with a median creatinine variation of − 17 (IQR 25–75 [− 55; +10.75]). Thirty-five patients received bisphosphonates with a median creatinine variation of − 51 (IQR 25–75 [− 139; +2.5]).

## Discussion

The present study is the first to describe etiological investigations and clinical course of ICU patients hospitalized for severe HCM. Indeed, previous studies have focused on the association between ionized calcemia and ICU mortality. Egi et al. [6] found an association between severe HCM and ICU mortality. Conversely, Zhang et al. [23] in a large multicentric cohort did not confirm this association. Beyond these epidemiological studies, there are no data focusing on clinical and biological characterization of these patients.

During HCM, one of the main reasons for ICU admission is the risk of cardiac rhythm disorders and Brugada-like electrocardiographic pattern. A recent study has effectively shown an association between HCM and shorter QT interval, longer PR interval, and J point elevation (mimicking a Brugada syndrome) regardless of the HCM etiology [24]. Brugada-type EKG is associated with increased risk of fatal ventricular arrhythmias and sudden death [25]. Only a few previous case reports studies described ventricular tachycardia and fibrillation in HCM patients [26, 27]. In our study, one patient presented with an ST segment elevation, and another patient (with concomitant hypokalemia) presented with a ventricular tachycardia. The scarcity of severe cardiac rhythm disorder found in our study may be explained by an early ICU admission policy in the two centers and an early treatment of HCM (with a median delay of bisphosphonate therapy on the day of admission).

Beside cardiovascular events, AKI was frequent and often severe (19% of AKI patients required renal replacement therapy). Multiple mechanisms may be involved in HCM-induced AKI, including a decrease of glomerular ultrafiltration coefficient [28], the induction of nephrogenic diabetes insipidus via down-regulation of aquaporin-2, disruption of countercurrent multiplier system [29, 30] and a loop diuretic-like effect [31], that participate to polyuria and volume depletion. Accordingly, almost two-third of HCM-induced AKI patients had a pre-renal AKI phenotype and 11% where polyuric prior ICU admission. Other factors, such as nephrotoxic drugs, tumor lysis syndrome, and an underlying hematological malignancy with renal involvement have participated to AKI episodes in our study. Our study was underpowered to show an impact of AKI on mortality. However, as small changes in serum creatinine have been shown to be associated with increased mortality [32, 33], prolonged hospital stay, and decrease of complete remission of the underlying malignancy in hematologic patients [34], we believe that prompt treatment of HCM to prevent AKI is of utmost importance in HCM patients.

In multivariate analysis, an underlying solid tumor was independently associated with hospital mortality. One explanation is that HCM is often a late complication in the course of solid tumors appearing in our study in 24% of cases in metastatic stage. Second, HCM patients with underlying solid tumors experienced more neurological complications. Delirium is known to be associated with longer hospital stay, higher ICU, and hospital mortality [35, 36]. We then believe that HCM patients with neurological symptoms require aggressive treatment of HCM.

ICU mortality in our cohort was 9.9%, which is consistent with the previous studies [37]. However, in the onco-hematology subgroup, 17.3% of ICU survivors died during hospitalization, after the correction of HCM. Causes of death are often multifactorial in these patients and HCM is mainly a marker of an advanced disease. These rates are far lower than usually reported in patients with onco-hematological malignancies hospitalized in ICU. Indeed, a prospective multicentric study grouping 1011 patients with hematological malignancies found an ICU and hospital mortality rate at 27.6% and 38.3%, respectively [38]. Even worse results were found in a Korean monocentric study gathering onco-hematological patients. ICU and hospital mortality rate were, respectively, 32.2% and 56% [39]. This discrepancy is explained by two reasons: first, the nature of ICU admission in our study. While the most common ICU admissions reasons are usually sepsis and acute respiratory failure, the main reason of admission in our study was the HCM itself without a significant rate of multivisceral failure, as suggested by the low median levels of SOFA scores at admission. Second, early ICU admission of these patients allowed prompt HCM aggressive therapy. Indeed, almost all patients received an adequate hyperhydration; 80% of them receiving bisphosphonate infusion at day 0. Only the use of bisphosphonate has been linked with a significant decrease of calcemia on day 5. We believe that this medication should be the cornerstone of the treatment of severe HCM, regardless of renal function.

Our study has several limitations. First, because of the retrospective design of the study, unidentified confounding factors may have been overlooked in the multivariable analysis. Second, due to the small number of patients in our cohort, our study may have been underpowered to show any relationship between HCM-induced AKI and mortality. Moreover, the results of our multivariate analysis show that our confidence intervals are wide, suggesting statistic instability. Third, the high prevalence of onco-hematological malignancies in our cohort may have introduced selection bias in our results. Indeed, this high prevalence may be explained by the specialized recruitment of onco-hematological patients in one center. However, this is consistent with the previous studies that have found malignancies as the main causes of HCM in emergency department [40].

## Conclusion

Finally, patients with severe HCM are at high risk of organ failures that are most of the time reversible with the early ICU management. An early aggressive therapy of HCM may prevent these complications, mainly AKI. A special attention should be paid to patients with onco-hematological malignancies to detect neurological complications associated with HCM.

Prospective studies are needed to finely evaluate the existence of a threshold of HCM beyond which hospitalization in intensive care is necessary or to identify the most effective treatments.

## Supplementary information

**Additional file 1: Figure S1.** Calcemia (mg/dL) course between day 0 and day 9. **Table S1.** Patients characteristics with suspected vitamin D intoxication. **Table S2.** Extra-renal manifestations. **Table S3.** Univariate analysis of determinants of complications of HCM. **Table S4.** Impact of therapies on total calcemia at day 5.

## Data Availability

The data sets used and analyzed during the current study are available from the corresponding author on reasonable request.

## Abbreviations

AKI: acute kidney injury
CKD: chronic kidney disease
eGFR: estimated glomerular filtration rate
HCM: hypercalcemia
ICU: intensive-care unit
IQR: interquartile range
OR: odds ratio
PTH: parathyroid hormone
PTH-rp: parathyroid hormone-related peptide
RRT: renal replacement therapy
SAPS II: simplified acute physiology score
Scr: serum creatinine
SOFA: sequential organ failure assessment
