# Supplementary material for: Epidemiology, clinical features, and management of severe hypercalcemia in critically ill patients
Source: Ann Intensive Care. 2019 Nov 27;9:133. doi: 10.1186/s13613-019-0606-8 (PMC6881488; doi:10.1186/s13613-019-0606-8)
Supplement: Supplementary file 1 — Additional file 1: Figure S1. Calcemia (mg/dL) course between day 0 and day 9. Table S1. Patients characteristics with suspected vitamin D intoxication. Table S2. Extra-renal manifestations. Table S3. Univariate analysis of determinants of complications of HCM. Table S4. Impact of therapies on total calcemia at day 5. [file 13613_2019_606_MOESM1_ESM.docx]

**Additional data**

**Table S1.** Patients characteristics with suspected vitamin D intoxication

| **Patients** | **Age** | **CKD** | **Thyroidectomy** | **Parathyroidectomy** | **Digestive disease** | **Calcium supplementation** | **1-25 (OH) supplementation** | **Cholecalciferol (25 (OH) supplementation)** | **PTH (pmol/L)** | **25 OH (pmol/L)** | **1-25 OH vit D (pmol/L)** |
| --- | --- | --- | --- | --- | --- | --- | --- | --- | --- | --- | --- |
| 1 | 67 | 1 | 1 | 0 (suspected) | 0 | 1 (2g/day) | 1 (2 µg/day) | 0 | 0 | 30,4 | 30,9 |
| 2 | 53 | 0 | 1 | 1 | 0 | 1 (1,54g/day) | 1 (3µg/day) | 0 | 0 | 22 | 41 |
| 3 | 56 | 1 | 1 | 1 | 0 | 1 (1,54g x3/day) | 1 (3µg/day) | 0 | 0 | 10 | NA |
| 4 | 69 | 0 | 1 | 1 | 0 | 1 (1g/day) | 1 (1µg/day) | 1 (45 mg/day) | 1,4 | > 125 | 39,7 |
| 5 | 69 | 0 | 0 | 0 | 0 | 1 (unknown) | 1 (unknown) | 0 | 1,9 | 24 | 30 |
| 6 | 56 | 0 | 0 | 0 | 0 | 1 (600 mg x2/day) | 1 (2µg/day) | 0 | 9 | 31,1 | 31 |
| 7 | 80 | 0 | 0 | 0 | 0 | 0 | 0 | 1 (unknown) | 25 | 724 | 67 |
| 8 | 76 | 0 | 1 | 1 | 0 | 1 (1,54 g x3/day) | 1 (0,5 µg/day) | 0 | < 5 | 18 | 62 |
| 9 | 44 | 0 | 1 | 0 | Crohn disease | 1 (600 mg x4/day) | 1 (2 µg/day) | 0 | < 5 | 65 | 66 |
| 10 | 25 | 0 | 1 | 0 | 0 | 1 (1 g x2/day) | 1 (2 µg/day) | 1 (unknown) | < 5 | 9 | < 7,5 |
| 11 | 72 | 0 | 1 | 0 | 0 | 1 (unknown) | 1 (0,75 µg/day) | 0 | <5 | 66,7 | 158 |
| 12 | 37 | 1 | 0 | 1 | 0 | 1 (1,54 g x2/day) | 1 (1 µg/day) | 0 | NA | NA | NA |
| 13 | 65 | 0 | 1 | 1 | 0 | 1 (1,54 g x6/day) | 1 (3 µg/day) | 0 | NA | NA | NA |
| 14 | 41 | 0 | 1 | 1 | 0 | 1 (unknown) | 1 (3 µg/day) | 0 | NA | 33 | 47 |
| 15 | 58 | 0 | 0 | 0 | Crohn disease | 1 (1,5 g/day) | 1 (2 µg/day) | 1 (unknown) | NA | NA | NA |

**Table S2.** Extra-renal manifestations

|  | **All (n = 131)** |
| --- | --- |
| ***Neurological manifestations, n (%)*** | 51 (38,9) |
| Glasgow score scale | 15 [14; 15] |
| Seizures, n (%) | 3 (2.3) |
| Delirium, n (%) | 39 (29.8) |
| ***Cardiovascular manifestations, n (%)*** | 73 (55.7) |
| Hypertension, n (%) | 23 (17.5) |
| Sinus tachycardia, n (%) | 31 (23.6) |
| Rhythm disturbances, n (%) | 5 (3.8) |
| Conduction disturbances, n (%) | 20 (15.2) |
| Negative T wave, n (%) | 10 (7.6) |
| Short QTc, n (%) | 19 (14.5) |
| Long QTc, n (%) | 7 (5.3) |
| ***Digestive manifestations, n (%)*** | 50 (38.1) |
| Abdominal pain, n (%) | 26 (19.8) |
| Constipation, n (%) | 24 (18.3) |
| Acute pancreatitis, n (%) | 5 (3.8) |
| Digestive occlusion, n (%) | 3 (2.3) |


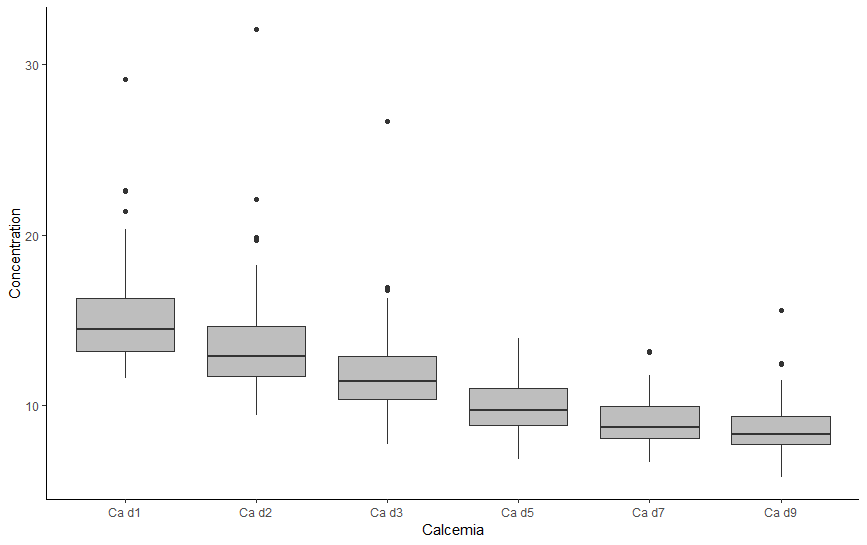


**Figure S1.** Calcemia (mg/dL) course between day 0 and day 9

**Table S3.** Univariate analysis of determinants of complications of HCM and hospital mortality

|  | **OR (95% IC)** | **P** |
| --- | --- | --- |
| ***Associated factors with cardiovascular complications*** |  |  |
| Age > 60 years | 1.54 (0.77; 3.08) | 0.23 |
| Male sex | 0.35 (0.17; 0.71) | 0.004 |
| Preexisting cardiopathy | 0.57 (0.26; 1.27) | 0.17 |
| Calcemia at day 1, per mmol/L | 1.98 (1.08; 3.64) | 0.028 |
| Etiologies |  |  |
| Other | 1 |  |
| Solid Tumors | 0.60 (0.21; 1.72) | 0.34 |
| Endocrinopathies | 0.93 (0.26; 3.31) | 0.91 |
| Hemopathies | 0.60 (0.24; 1.51) | 0.27 |
| ***Associated factors with neurological complications*** |  |  |
| Age > 60 years | 1.45 (0.71; 2.93) | 0.31 |
| Male sex | 1.28 (0.63; 2.59) | 0.50 |
| Calcemia at day 1, per mmol/L | 1.72 (0.99; 2.98) | 0.05 |
| Etiologies |  |  |
| Other | 1 |  |
| Solid Tumors | 9.62 (2.85; 32.5) | 0.003 |
| Endocrinopathies | 0.52 (0.09; 2.97) | 0.47 |
| Hemopathies | 2.24 (0.79; 6.38) | 0.13 |
| ***Associated factors with AKI stage > 2^a^*** |  |  |
| Age > 60 years | 0.38 (0.08; 1.69) | 0.20 |
| Male sex | 3.69 (0.65; 21.0) | 0.14 |
| Calcemia at day 1, per mmol/L | 0.48 (0.07; 3.39) | 0.50 |
| Etiologies |  |  |
| Hemopathies | 1 |  |
| Solid Tumors | 0.14 (0.013; 1.63) | 0.10 |
| Endocrinopathies | 0.10 (0,009; 1.10) | 0.06 |
| Others | 1.00 (0.13; 7.57) | 1 |
| Chronic kidney disease | 1.31 (0.27; 6.37) | 0.70 |
| ***Associated factors with hospital mortality*** |  |  |
| Calcemia at day 1, per mmol/L | 1.26 (0.05; 25) | 0.51 |
| Etiologies |  |  |
| Others | 1 |  |
| Solid Tumors | 11.13 (1.92; 112.6) | 0.02 |
| Endocrinopathies | 1.43 (0.05; 24.9) | 0.80 |
| Hemopathies | 2.79 (0.54; 24.4) | 0.27 |
| Neurological complications | 3.32 (1.15; 10.1) | 0.03 |
| Cardiovascular complications | 1.08 (0.38; 3.1) | 0.88 |
| AKI stage > 1 | 0.58 (0.17; 2.06) | 0.38 |

Abbreviations: AKI, acute kidney injury; CI, confidence interval; OR, odds ratio.

^a^For AKI stage >2, 3 patients with chronic kidney disease were excluded due to preexisting dialysis treatment.

**Table S4.** Impact of therapies on total calcemia at day 5.

|  | **HR (95% IC)** | **P** |
| --- | --- | --- |
| Corticosteroids | 0.86 (0.58; 1.27) | 0.45 |
| Furosemide | 0.94 (0.48; 1.87) | 0.87 |
| Bisphosphonate | 0.42 (0.27; 0.67) | < 0.001 |
| Salmon calcitonin | 0.63 (0.19; 2.01) | 0.43 |
